# Global, regional, and national burden of fracture of pelvis, 1990–2021: analysis of data from the Global Burden of Disease Study 2021

**DOI:** 10.3389/fpubh.2025.1610604

**Published:** 2025-06-18

**Authors:** Jiyong Wei, Dezhi He, Guipeng Lan, Minglian Xu, Jianfeng Guo, Yanni Lan, Shaohui Zong

**Affiliations:** ^1^Department of Spine Osteopathia, The First Affiliated Hospital of Guangxi Medical University, Nanning, Guangxi, China; ^2^Department of Spine Surgery, The First People's Hospital of Nanning, The Fifth Affiliated Hospital of Guangxi Medical University, Nanning, Guangxi, China; ^3^Guangxi Zhuang Autonomous Region Institute for the Prevention and Treatment of Occupational Disease, Nanning, Guangxi, China; ^4^Department of Trauma Orthopedics, The Eighth People's Hospital of Nanning, Nanning, Guangxi, China; ^5^Department of Orthopedics Trauma and Hand Surgery, The First Affiliated Hospital of Guangxi Medical University, Nanning, Guangxi, China; ^6^Department of Pharmacy, The People's Hospital of Guangxi Zhuang Autonomous Region and Guangxi Academy of Medical Sciences, Nanning, Guangxi, China; ^7^Wuming Hospital of Guangxi Medical University, Nanning, Guangxi, China

**Keywords:** fracture of pelvis, GBD, incidence, prevalence, YLDs

## Abstract

**Background:**

Fractures of the pelvis are significant orthopedic injuries associated with high morbidity, mortality, and substantial economic burden worldwide.

**Methods:**

This study aimed to comprehensively analyze the disease burden of pelvic fractures globally from 1990 to 2021 using data from the Global Burden of Disease (GBD) Study 2021. First, we conducted a descriptive analysis in 2021, stratifying data by subtypes. Secondly, we used the Linear regression models to analyze temporal trends. Finally, we used two models to predict the future burden. Furthermore, we examined the correlation between estimated annual percentage change (EAPCs) and age-standardized rates (ASRs), as well as Human Development Index (HDI) scores in 2021.

**Results:**

In 2021, pelvic fractures caused 4,524,448 incident cases (95% UI 3,283,345–6,583,735), 13,100,257 prevalent cases (12,103,233–14,174,613), and 2,241,606 years lived with disability (YLDs; 1,559,349–2,965,288). The age-standardized incidence rate (ASIR) was 56.00 per 100,000 (40.96–81.22), age-standardized prevalence rate (ASPR) 155.97 (143.85–168.87), and YLDs rate 26.74 (18.59–35.36). Rates were higher in males, with ASIR, ASPR, and YLDs 1.14, 1.35, and 1.37 times those in females. Age-specific ASRs rose with age. Australasia had the highest ASIR (148.39; 101.02–219.91), and the Commonwealth Low-Income region the lowest (21.97; 16.86–29.23). Western Africa recorded the lowest ASPR (77.37; 70.44–86.42) and YLD rate (13.34; 9.39–18.00). Nationally, ASIR was highest in Andorra (176.62; 111.81–281.70) and lowest in Kiribati (17.96; 13.88–23.10). Projections suggest rising burden through 2046. EAPCs were inversely associated with ASRs and HDI, except for a weak, non-significant positive correlation with ASIR (ρ = 0.08; *P* = 0.27).

**Conclusion:**

Our findings reveal a substantial and increasing global burden of pelvic fractures, particularly in regions with limited access to high-quality trauma care. The increasing proportion of years lived with disability (YLDs) due to long-term disability underscores the importance of comprehensive management strategies, including prevention, timely treatment, and effective rehabilitation.

## 1 Introduction

Fractures of the pelvis are a significant health concern globally, accounting for a substantial proportion of trauma-related morbidity and mortality. These injuries are often the result of high-energy trauma, such as road traffic accidents, falls, and industrial accidents, and can lead to severe long-term disabilities, including chronic pain, mobility impairments, and psychological distress (1, 2). Understanding the global burden of pelvic fractures is crucial for the effective allocation of healthcare resources and the development of targeted interventions to reduce their impact.

Previous studies have examined the incidence and outcomes of pelvic fractures in specific regions or countries, revealing significant variations in disease burden (3–5). However, a comprehensive analysis of the global burden of pelvic fractures over an extended period is lacking. The Global Burden of Disease (GBD) Study provides a unique opportunity to fill this gap by offering a standardized and comprehensive framework for assessing the health impact of various diseases and injuries across different populations and time points (6).

The GBD Study uses a combination of epidemiological data, demographic information, and health outcome measures to estimate the burden of diseases and injuries. This approach allows for the comparison of disease burden across diverse populations and the identification of trends over time (7). Previous GBD studies have focused on various health conditions, including musculoskeletal disorders, but few have specifically addressed pelvic fractures (8–10).

In this study, we aim to analyze the global burden of pelvic fractures from 1990 to 2021 using data from the GBD Study 2021. Our objectives are to estimate the number of incidence cases, the number of prevalence cases, the number of years lived with disability (YLDs) cases, and the corresponding age-standardized rates (ASRs) for pelvic fractures across sexes, age groups, sociodemographic index (SDI) regions, GBD regions, and countries. By doing so, we hope to provide a comprehensive overview of the global burden of pelvic fractures and identify key trends and patterns that can inform future research and policy decisions.

Several studies have reported on the incidence and outcomes of pelvic fractures in specific settings. For example, a study in the United States found that pelvic fractures are associated with high mortality and substantial long-term disability (3). Similarly, a study in China reported a significant increase in the incidence of pelvic fractures over a 10-year period, with younger age groups being most affected (4). However, these studies are limited to specific regions and may not reflect the global burden of pelvic fractures.

In contrast, the GBD Study offers a global perspective, allowing for the comparison of disease burden across different populations and regions. Previous GBD studies have demonstrated the value of this approach in understanding the global burden of various health conditions, including musculoskeletal disorders (6, 8, 9). By extending this framework to pelvic fractures, we aim to provide a more comprehensive understanding of their global impact.

## 2 Methods

### 2.1 Introduction to study design and data sources

The present study aims to comprehensively assess the disease burden of fracture of the pelvis globally from 1990 to 2021, utilizing data from the GBD Study 2021. The GBD Study is a collaborative effort involving hundreds of researchers worldwide, aimed at providing a comprehensive and up-to-date assessment of the health status of populations across various diseases and injuries (10). Data for this analysis were sourced from multiple repositories, including vital registration systems, surveys, and hospital records, which were meticulously curated and standardized to ensure consistency and comparability across different regions and time points (11).

### 2.2 Estimation of incidence and deaths rate

The incidence and deaths rates from the GBD 2021 study of pelvic fractures were estimated using the DisMod-MR 2.1 tool, a Bayesian meta-regression model developed by the Institute for Health Metrics and Evaluation (IHME) (12). This tool allowed for the pooling of data from various sources, adjusting for potential biases and inconsistencies. Age-sex-specific and country-specific estimates were generated by fitting a series of mathematical models to the available data, incorporating covariates such as SDI, urbanization, and health system performance (13).

### 2.3 Calculation of YLDs

YLDs were calculated using disability weights derived from population-based surveys, which reflect the societal perception of the severity of health loss associated with specific health outcomes (7). For pelvic fractures, disability weights were sourced from the most recent GBD study and adjusted for long-term sequelae such as chronic pain, mobility impairments, and the need for long-term rehabilitation (14).

### 2.4 Statistical analysis

Initially, we conducted a descriptive analysis utilizing the GBD 2021 database, specifically focusing on data pertaining to the year 2021. In this analysis, we stratified the data into multiple subgroups, including sex, age, SDI regions, GBD regions, and individual countries.

Subsequently, we analyzed temporal trends in the fracture of the pelvis-related burden spanning from 1990 to 2021. To quantify these trends, we employed linear regression models to estimate the Estimated Annual Percentage Change (EAPC) values for all indicators of disease burden at the global level and across various subgroups. Positive EAPC values indicated an increasing trend, whereas negative values denoted a decreasing trend. Additionally, to investigate the patterns of disease burden across GBD regions, we performed a cluster analysis using the EAPC values for all indicators.

To enhance the robustness of future burden estimates, we employed both the classical age–period–cohort (APC) model and the Bayesian age–period–cohort (BAPC) model, which capture temporal patterns across age, period, and cohort dimensions. The BAPC model further incorporates prior distributions and explicitly quantifies uncertainty, enabling more stable and reliable projections in the presence of data fluctuations.

Finally, to explore potential associations between EAPCs and socioeconomic factors, we examined the correlation between EAPCs and fracture of the pelvis-related ASRs, as well as Human Development Index (HDI) scores in 2021. Given the non-normal distribution of these variables, we adopted the Spearman correlation analysis, a non-parametric statistical method, to assess the strength and direction of these associations.

All statistical analyses were executed using R version 4.0.3 (R Foundation for Statistical Computing, Vienna, Austria).

## 3 Results

### 3.1 Disease burden of pelvic fractures in 2021

In the year 2021, pelvic fractures accounted for 4,524,448 incident cases, with a 95% uncertainty interval (UI) ranging from 3,283,345 to 6,583,735. The corresponding age-standardized incidence rate (ASIR) was 56.00 per 100,000 population, with a 95% UI of 40.96 to 81.22. Additionally, the prevalence of pelvic fractures amounted to 13,100,257 cases, with a 95% UI of 12,103,233 to 14,174,613, and the age-standardized prevalence rate (ASPR) was 155.97 per 100,000 population, with a 95% UI of 143.85 to 168.87. Furthermore, the total number of YLDs due to pelvic fractures was 2,241,606, with a 95% UI of 1,559,349 to 2,965,288. The corresponding ASR of YLDs was 26.74 per 100,000 population, with a 95% UI of 18.59 to 35.36 (Supplementary Tables 1–3).

Male individuals exhibited a slightly higher burden of pelvic fractures compared to females in 2021. Specifically, the number of incident cases was 1.07 times greater in males, the number of prevalence cases was 1.28 times higher, and the number of YLDs cases was 1.29 times greater. Similarly, the corresponding ASRs were 1.14 times higher for incidence, 1.35 times higher for prevalence, and 1.37 times higher for YLDs in males compared to females (Supplementary Figure S1, Supplementary Tables 1–3).

The distribution of incidence, prevalence, and YLDs across different age groups in 2021 for pelvic fractures revealed a distinct pattern. Initially, the number of incident, prevalent, and YLDs cases increased with age, reaching a peak, and then subsequently declined. However, for the corresponding ASRs, the disease burden increased with age (Supplementary Figure S2, Supplementary Tables 1–3).

**Figure 1:**
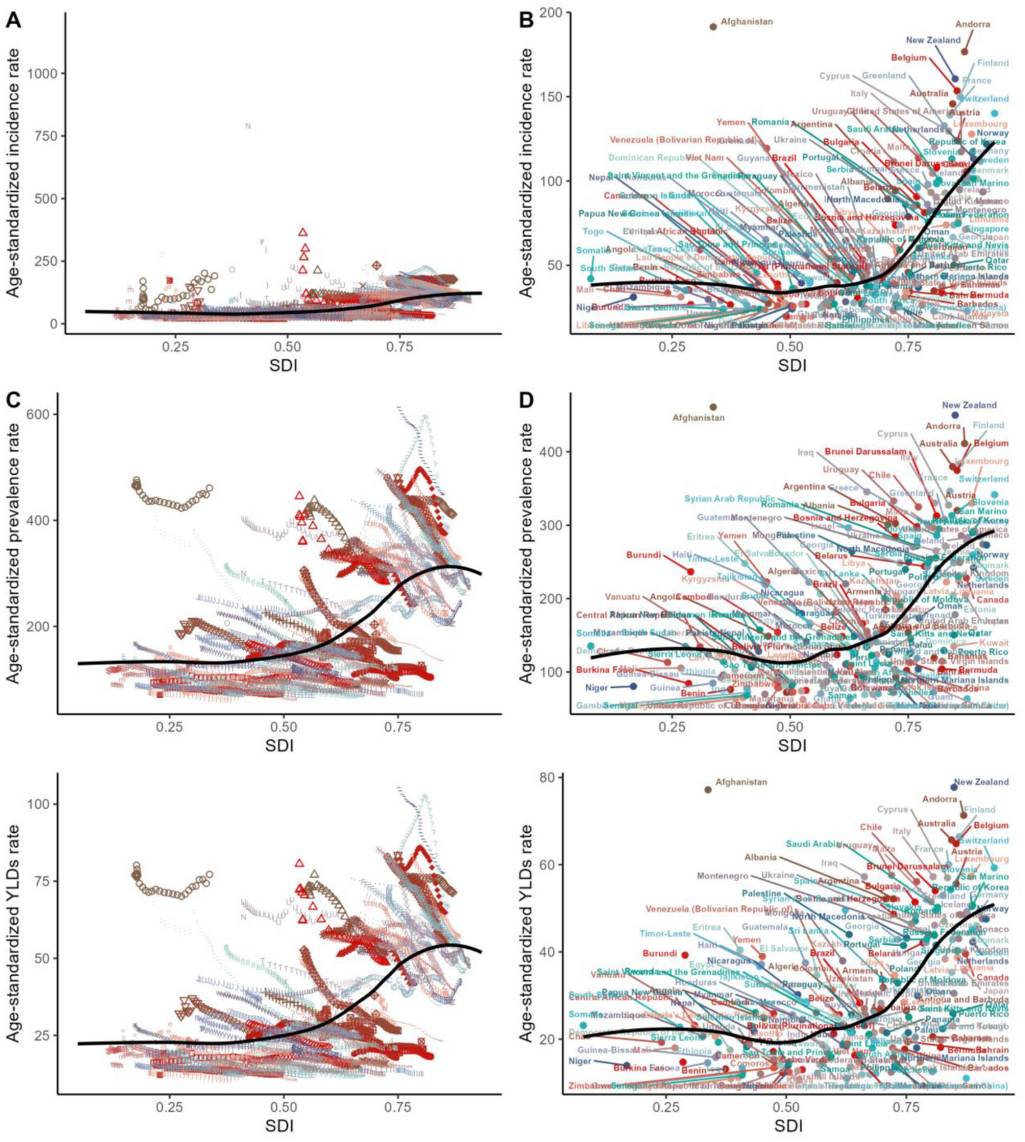
Age-standardized rates of incidence, prevalence, and YLDs of fracture of pelvis across countries and territories by socio-demographic index for both sexes, 1990–2021. The black line was an adaptive association fitted with adaptive Loess regression based on all data points. Different images and symbols represent different countries. YLDs, years lived with disability.

At the level of the SDI regions, the number of cases and corresponding ASRs associated with pelvic fractures for incidence, prevalence, and YLDs exhibited a “J-shaped” relationship with increasing SDI. Specifically, the burden was highest in high SDI regions (Figure 1, Supplementary Figure S3, Supplementary Tables 1–3).

Among the 54 GBD regions, Asia ranked first in the number of pelvic fracture-related cases, followed by Advanced Health System and World Bank High Income regions. Oceania ranked last in the number of cases, followed by Southern Sub-Saharan Africa. For the corresponding ASRs, Australasia ranked first, while Commonwealth Low Income regions ranked last for ASIR, and Western Africa ranked last for ASRs of prevalence and YLDs (Supplementary Figure S4, Supplementary Tables 1–3).

**Figure 2:**
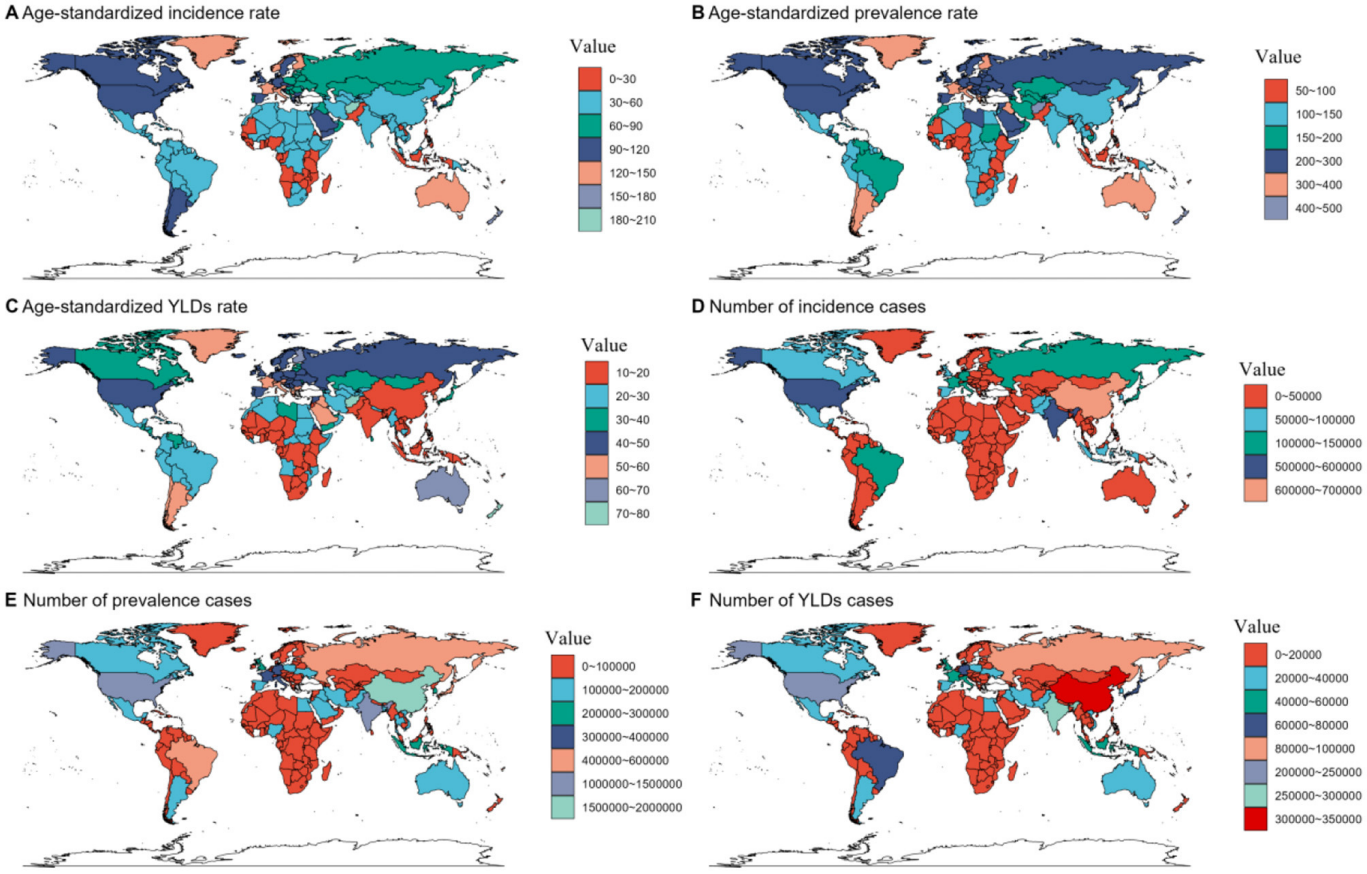
Numbers and age-standardized rates of fracture of pelvis-related incidence, prevalence, and YLDs across countries and territories in 2021. YLDs, disability-adjusted life years.

The disease burden of pelvic fractures varied significantly across the world. China had the highest number of cases, followed by India and the United States of America. Tokelau and Niue had the lowest number of incident, prevalent, and YLDs cases. As for the ASRs, Andorra had the highest ASRs, while Kiribati had the lowest ASRs (Figure 2, Supplementary Tables 1–3).

### 3.2 Temporal trend for the disease burden of pelvic fractures from 1990 to 2021

Between 1990 and 2021, the incidence, prevalence, and YLDs due to pelvic fractures exhibited an upward trajectory. Specifically, the incidence cases rose from 3,604,239 [95% uncertainty interval (UI): 2,794,736–4,801,902] to 4,524,448 [95% UI: 3,283,345–6,583,735]. Similarly, the prevalence cases increased from 10,109,439 (95% UI: 9,409,071–10,834,457) to 13,100,257 (95% UI: 12,103,233–14,174,613). Additionally, the number of YLDs cases increased from 1,752,618 (95% UI: 1,213,438–2,342,291) to 2,241,606 (95% UI: 1,559,349–2,965,288). However, the ASRs demonstrated a distinct downward trend, with EAPCs of −0.86 (95% confidence interval [CI]:−0.92 to−0.81) for incidence, −1.24 (95% CI: −1.29 to −1.20) for prevalence, and −1.24 (95% CI: −1.28 to −1.20) for YLDs (Supplementary Figure S5, Supplementary Tables 1–3).

The trends observed in both the number of cases and the ASRs for males and females were consistent with those of the overall population (Supplementary Figure S6, Supplementary Tables 1–3). These trends were also consistent across all age groups, except for the older adults in the ASIR indicator, as illustrated in Supplementary Figure S7 and supported by the data presented in Supplementary Tables 1–3. At the regional level of the SDI, the trends in the number of cases and the corresponding ASRs for incidence, prevalence, and YLDs of pelvic fractures mirrored the overall trend (Supplementary Figure S8, Supplementary Tables 1–3).

**Figure 3:**
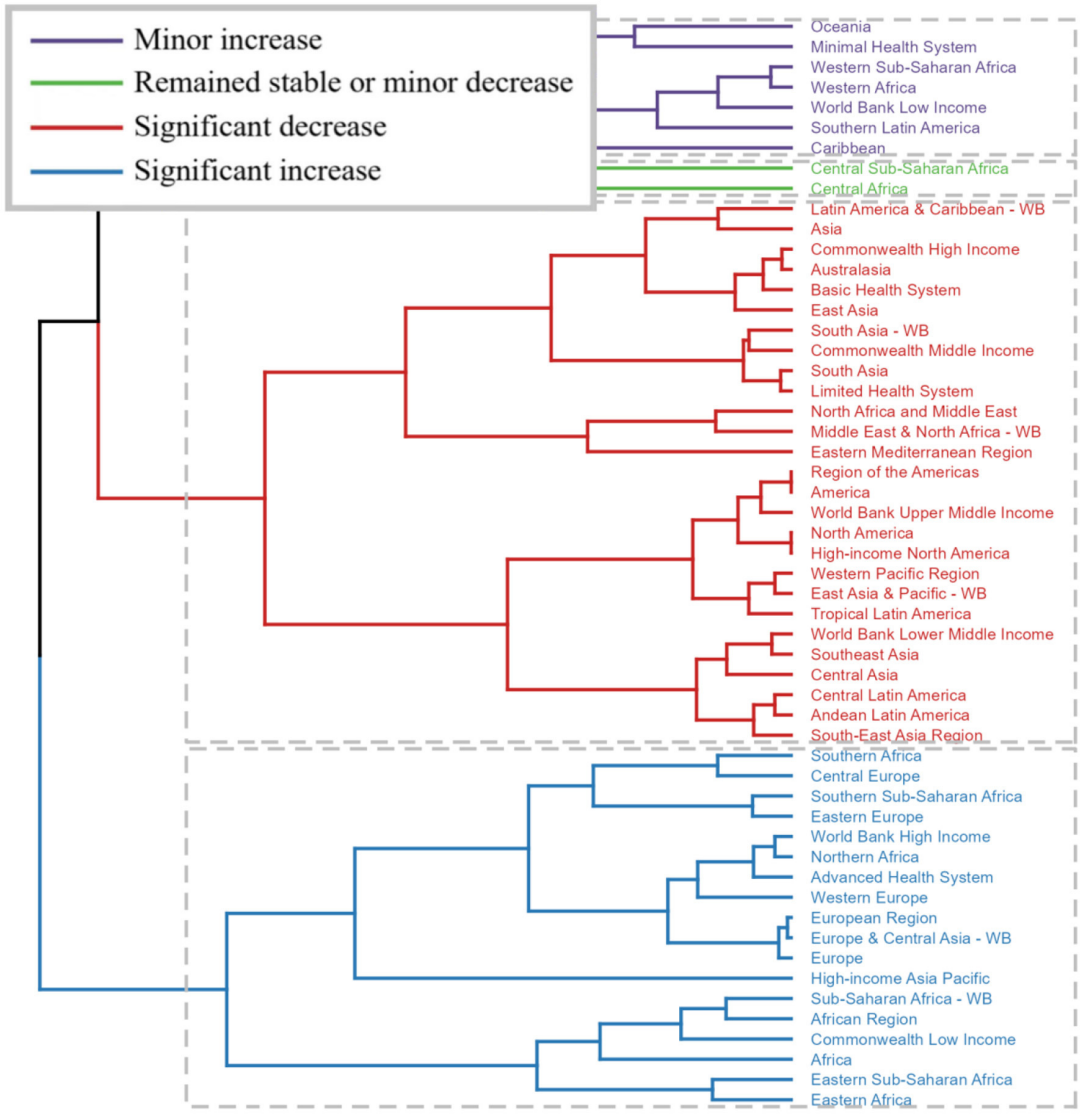
Results of cluster analysis based on the EAPC values of the age-standardized incidence, prevalence, and YLDs rates of fracture of pelvis from 1990 to 2021. EAPC, estimated annual percentage change; YLDs, years lived with disability.

Significant variations in the burden of pelvic fractures were observed across the various GBD regions. To identify regions with similar patterns of variation in disease burden, a hierarchical clustering analysis was conducted. The results of this analysis are presented in Figure 3. Regions such as Southern Africa, Central Europe, Southern Sub-Saharan Africa, Eastern Europe, World Bank High-income, Northern Africa, Advanced Health System, Western Europe, European Region, Europe and Central Asia -WB, Europe, High-income Asia Pacific, Sub-Saharan Africa -WB, African Region, Commonwealth Low-income, Africa, Eastern Sub-Saharan Africa, and Eastern Africa exhibited a significant increase in the incidence rate, prevalence rate, and YLDs rate. Conversely, a significant decrease was observed in 27 regions, including Latin America and Caribbean -WB, Asia, Commonwealth High-income, Australasia, Basic Health System, East Asia, South Asia -WB, Commonwealth Middle-income, South Asia, Limited Health System, North Africa and Middle East, Middle East and North Africa -WB, Eastern Mediterranean Region, Region of the Americas, America, World Bank Upper Middle-income, North America, High-income North America, Western Pacific Region, East Asia and Pacific -WB, Tropical Latin America, World Bank Lower Middle-income, Southeast Asia, Central Asia, Central Latin America, Andean Latin America, and South-East Asia Region (Figure 3, Supplementary Tables 1–3).

At the country and territory level, the trends also varied. The most notable decrease in ASIR (EAPC = −6.24, 95% CI: −8.01 to −4.43) from 1990 to 2021 was observed in Timor-Leste, while the most significant decrease in ASPR (EAPC = −2.59, 95% CI: −2.70 to −2.49) was seen in Portugal. The most pronounced decrease in the age-standardized rate of YLDs (EAPC = −2.59, 95% CI: −2.74 to −2.43) during the same period was observed in Taiwan (Province of China). Conversely, the most significant increase in ASIR (EAPC = 7.05, 95% CI: 4.44–9.73) was in the Syrian Arab Republic, the most pronounced increase in ASPR (EAPC = 2.91, 95% CI: 1.89-3.95) was in Burundi, and the most notable increase in the age-standardized rate of YLDs (EAPC = 2.87, 95% CI: 2.02–3.74) was also in the Syrian Arab Republic (Supplementary Figure S9, Supplementary Tables 1–3).

### 3.3 Projected outcomes from 2022 to 2046

**Figure 4:**
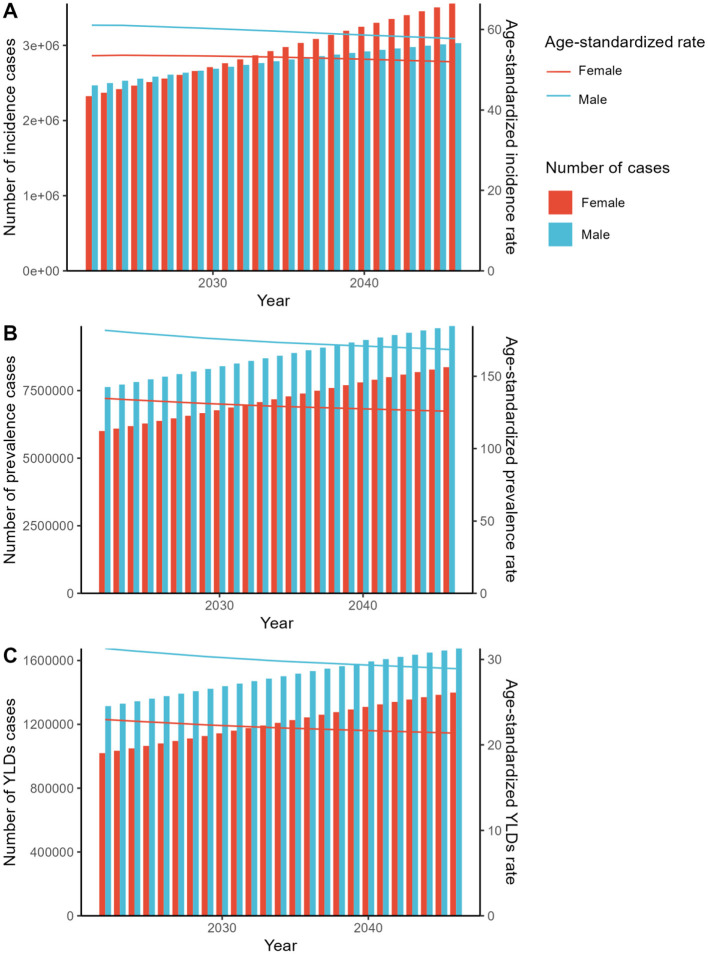
The predicted results in the fracture of pelvis-related numbers and age-standardized rates of incidence, prevalence, and YLDs by sex globally from 2022 to 2046 of the ARIMA model. YLDs, disability-adjusted-life-year; ARIMA, Autoregressive Integrated Moving Average.

**Figure 5:**
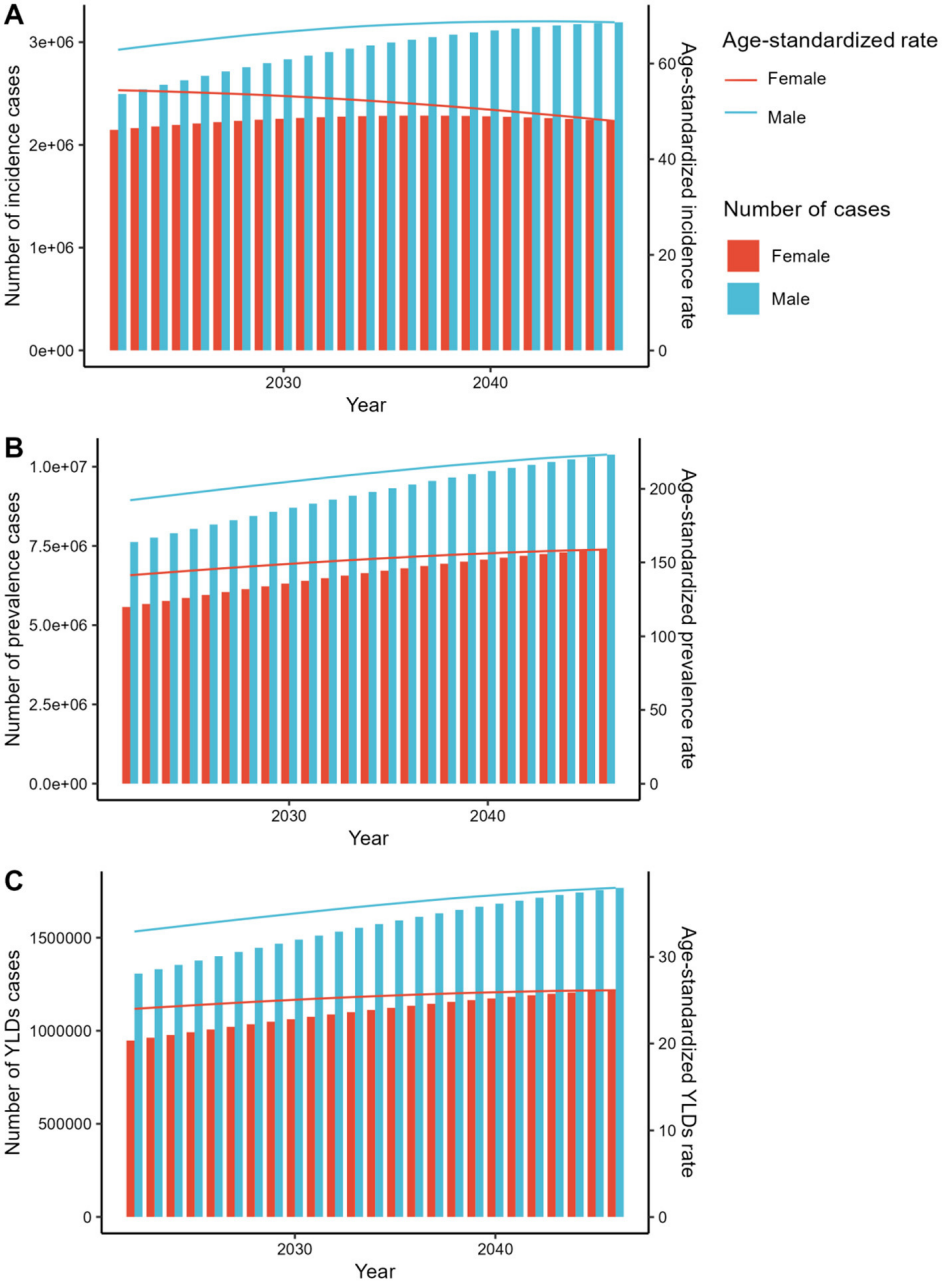
The predicted results in the fracture of pelvis-related numbers and age-standardized rates of incidence, prevalence, and YLDs by sex globally from 2022 to 2046 of the ES model. YLDs, disability-adjusted-life-year; ES, Exponential Smoothing.

The forecasted results derived from both the APC and BAPC models consistently reveal an upward trajectory in the number of incidence, prevalence, and YLDs cases. When analyzing the corresponding ASRs for both genders from 2022 to 2046, the APC model exhibits a declining trend. Conversely, in the BAPC model, with the exception of the ASIR for females, all indicators for both genders demonstrate an increasing pattern (Figures 4, 5, Supplementary Tables 4, 5).

### 3.4 Analysis of influential factors on the EAPC

**Figure 6:**
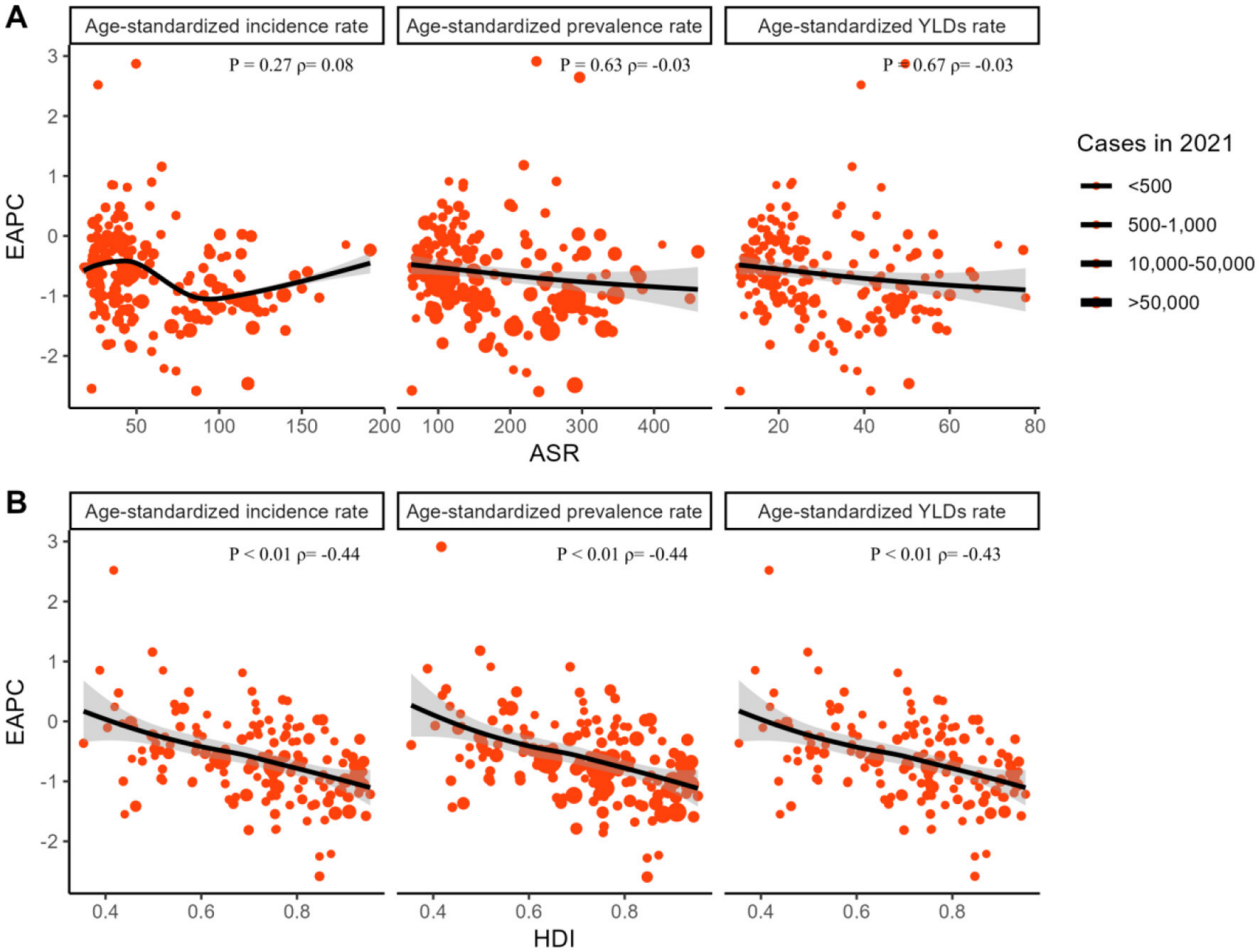
The association between EAPCs and fracture of pelvis-related ASRs and HDIs in 2021. The circles represent countries that were available on HDI data. The size of the circle is increased with the cases of fracture of pelvis. The ρ indices and *p*-values presented were derived from Spearman correlation analysis. EAPC, estimated annual percentage change; ASR, age-standardized rate; HDI, human development index.

Our findings demonstrate a notable correlation between EAPCs and the ASRs of pelvic fracture, as well as HDIs in 2021 (Figure 6). Specifically, the 2021 ASRs for pelvic fracture reflect the baseline disease burden, whereas the 2021 HDIs serve as proxies for healthcare accessibility and a marker of health system maturity within each country. A positive correlation is observed between EAPCs and ASRs for incidence (*P* = 0.27, correlation coefficient ρ = 0.08). However, for ASRs of prevalence (*P* = 0.63, ρ = −0.03) and YLDs (*P* = 0.67, ρ = −0.03), the association is negative yet nonsignificant. Furthermore, statistical correlations are identified between EAPCs for incidence, prevalence, and YLDs and HDIs, with negative correlations observed for incidence (*P* < 0.01, ρ = −0.44), prevalence (*P* < 0.01, ρ = −0.44), and YLDs (*P* < 0.01, ρ = −0.43) of pelvic fracture with the corresponding HDIs (Figure 6).

## 4 Discussion

This study disclosed the magnitude and temporal trends of pelvic fractures-related burden during the past three decades based on the lasted GBD 2021 and also conducted the projection until 2046. It revealed that the absolute number of pelvic fractures globally accounted for a relatively high proportion of the global numbers in 2021. As measured by trends, the number of cases attributable to pelvic fractures significantly increased from 1990 to 2021, and will still increasing in the future.

The findings of this study indicate a substantial burden of pelvic fractures globally, with notable trends observed between 1990 and 2021. The incident cases and prevalence of pelvic fractures have increased, highlighting a growing public health concern. This upsurge aligns with previous studies that have reported an increase in trauma-related injuries due to road traffic accidents, falls, and other causes (15). However, despite this rising absolute number of cases, the ASRs for incidence, prevalence, and YLDs due to pelvic fractures exhibited a decline. This downward trend in ASRs suggests potential improvements in healthcare delivery, such as enhanced trauma care systems and advancements in surgical techniques for pelvic fracture management (16, 17). The total YLDs due to pelvic fractures also increased over this period, indicating a significant impact on the quality of life of those affected. This increase in YLDs underscores the need for comprehensive rehabilitation programs and long-term care to mitigate the disability associated with these injuries (18). The observed decreasing trend in the ASR of YLDs may reflect improvements in rehabilitation services and patient outcomes following pelvic fracture treatment. When compared to existing literature, our study provides updated estimates with broader uncertainty intervals, reflecting the inherent challenges in gathering comprehensive global data on injury incidence and prevalence (10, 19). The decline in ASRs, despite rising incident and prevalent cases, necessitates further investigation into the factors driving these trends, such as changes in population demographics, health policies, and access to healthcare services. Understanding these dynamics will be crucial for developing targeted interventions to reduce the global burden of pelvic fractures.

Our findings reveal a slightly elevated burden of pelvic fractures among male individuals compared to females in 2021, which concurs with previous studies documenting gender-specific disparities in orthopedic trauma (20, 21). The observed higher incidence, prevalence, and YLDs among males, as indicated by ratios exceeding those of females, are in line with literature suggesting that males may be more predisposed to high-impact injuries due to occupational hazards, engagement in riskier activities, and potentially differences in bone density and structural integrity (22, 23). Notably, the consistency in trends between case numbers and ASRs for both genders mirrors those observed in the overall population, highlighting the persistence of this gender gap across different demographic metrics (24). These findings underscore the importance of gender-specific considerations in preventive strategies and clinical management to address the disproportionate burden of pelvic fractures in males. Future research should explore the underlying causes of these disparities in greater depth and evaluate the effectiveness of tailored interventions aimed at reducing the incidence and impact of pelvic fractures in males.

Our study observed a distinct age-related pattern in the incidence, prevalence, and YLDs of pelvic fractures in 2021, which aligns with previous literature indicating an age-dependent increase in orthopedic trauma (25, 26). Notably, while the number of incident, prevalent, and YLDs cases peaked at a certain age and subsequently declined, the ASRs consistently increased with age, suggesting that the absolute burden decreases with advancing age, but the relative risk per capita remains elevated (27). This discrepancy may be attributed to the competing risk of mortality among the oldest age groups, where despite a higher prevalence of fractures, fewer individuals survive to accumulate disability years (28). Consistently across age groups, the trends were maintained, except for a slight deviation in the ASIR among older adults, possibly due to variations in fall prevention measures and healthcare access (29). Overall, these findings emphasize the need for age-specific interventions and highlight the importance of considering population dynamics when planning orthopedic healthcare strategies.

In our study, the distribution of pelvic fracture cases, along with their incidence, prevalence, and YLDs, exhibited a “J-shaped” relationship with increasing SDI, peaking in high SDI regions. This finding aligns with previous literature indicating that while middle-SDI countries may face rising injury burdens due to rapid urbanization and industrialization, high-SDI regions continue to grapple with substantial healthcare challenges related to traumatic injuries, including pelvic fractures (15, 30). The regional-level trends mirrored the overall pattern, suggesting a consistent disparity across different development strata. The concentration of pelvic fracture burdens in high SDI areas could be attributed to aging populations and high-energy trauma incidents common in these regions (31, 32). Thus, tailored interventions focusing on injury prevention, particularly in high SDI settings, are crucial to mitigating the impact of pelvic fractures globally. Future research should explore the underlying factors contributing to this disparity further and evaluate the effectiveness of intervention strategies.

The present study highlights substantial disparities in the burden of pelvic fractures among various GBD regions. Asia emerged as the epicenter with the highest number of pelvic fracture-related cases, closely followed by Advanced Health System and World Bank High-income regions, aligning with previous reports indicating high trauma incidence in rapidly developing Asian countries (15, 30). Conversely, Oceania and Southern Sub-Saharan Africa reported the lowest case counts, suggesting potential differences in trauma exposure and healthcare access. When examining ASRs, Australasia led in incidence, whereas the Commonwealth Low-income regions lagged behind in ASIR, and Western Africa exhibited the lowest ASRs for prevalence and YLDs. Our hierarchical clustering analysis further delineated distinct regional patterns. Notably, several regions in Africa, Europe, and Asia Pacific demonstrated notable increases in incidence, prevalence, and YLDs rates, possibly attributed to population growth, aging demographics, and evolving trauma epidemiology (33). In stark contrast, 27 regions, predominantly in Latin America, Caribbean, Asia, and parts of the Middle East, exhibited decreases, which could be linked to advancements in trauma care, injury prevention measures, and socio-economic developments (14, 22). These findings underscore the multifaceted nature of pelvic fracture epidemiology, emphasizing the need for tailored prevention strategies and resource allocation across diverse global settings.

The global burden of pelvic fractures exhibits considerable variation, with China, India, and the USA topping the list of most affected countries, while smaller islands such as Tokelau and Niue report the least incident, prevalent, and YLDs cases. At the country level, the trends in pelvic fracture incidence and burden are equally diverse. Notably, Timor-Leste showed a substantial decrease in ASIR, potentially reflecting advancements in trauma care and preventive measures (34). Portugal's notable decline in ASPR may be attributed to improvements in healthcare delivery and patient management strategies (35). Taiwan (Province of China) demonstrated a marked reduction in the age-standardized rate of YLDs, suggesting effective rehabilitation services and timely interventions post-injury (36). Conversely, Syria and Burundi exhibited significant increases in ASIR, ASPR, and YLDs, respectively, which could be linked to ongoing conflicts, socio-economic disruptions, and inadequate healthcare infrastructure (37, 38). These findings underscore the complex interplay of socio-economic, demographic, and healthcare factors influencing pelvic fracture epidemiology worldwide and emphasize the need for tailored intervention strategies in different regions.

The projected outcomes from 2022 to 2046 for incidence, prevalence, and YLDs cases indicate a consistent upward trajectory across both the APC and BAPC models, suggesting a growing burden of the condition globally. This aligns with previous studies that have reported increasing trends in chronic disease burdens (19, 39). However, when examining the ASRs, the APC model shows a declining trend, which could be attributed to population aging and improvements in healthcare delivery and disease management (40). In contrast, the BAPC model reveals an increasing pattern for most indicators, with the exception of the ASIR for females, which may reflect gender-specific differences in risk factors and access to healthcare services. These discrepancies between models highlight the complexity of disease burden projections and the need for comprehensive and multifaceted approaches to address the underlying drivers of disease incidence and progression.

Our analysis of influential factors on the EAPCs highlights intriguing correlations between EAPCs and both pelvic fracture ASRs and HDIs in 2021. The baseline disease burden, represented by pelvic fracture ASRs in 2021, appears to exhibit a weak positive correlation with EAPCs for incidence, although this is not statistically significant. This aligns with previous studies indicating that higher baseline disease burdens may influence the rate of change over time (39). Conversely, the negative yet non-significant correlations observed between EAPCs for prevalence and YLDs and their respective ASRs suggest more complex relationships that may be influenced by factors such as improvements in healthcare delivery and population aging (40). Notably, the strong negative correlations between EAPCs for incidence, prevalence, and YLDs and HDIs indicate that countries with higher healthcare accessibility and more mature health systems exhibit slower rates of increase in pelvic fracture burden. This finding is consistent with studies showing that healthcare advancements can mitigate the impact of chronic conditions (10). Further research is needed to explore these relationships in greater depth and to identify potential interventions that could effectively reduce the burden of pelvic fractures globally.

Despite prior research highlighting the constraints associated with the GBD studies (41–43), it remains imperative to delineate the specific limitations pertinent to our work. Firstly, the analysis of the burden of pelvic fractures in this study may be constrained by the limited detailed information available from the GBD database. Notably, while the GBD 2021 study introduced refinements to its model to enhance estimation precision, these enhancements do not fully mitigate the informational constraints faced in our analysis. Furthermore, our study endeavors to assess evolving trends and forecast the burden of pelvic fractures based on the GBD 2021 data. However, the inherent information lag within this database, which currently restricts data access to the period from 1990 to 2021, could potentially undermine the accuracy of our predictive outcomes. Nevertheless, our findings hold substantial public health significance, contributing valuable insights for the global management and mitigation of the burden related to pelvic fractures.

## 5 Conclusion

Our study provided a comprehensive analysis of the global, regional, and national burden of pelvic fracture incidence, prevalence, and YLDs. The findings underscored the need for targeted interventions to reduce the burden of pelvic fracture globally. Future research should focus on identifying the drivers of these trends and evaluating the effectiveness of interventions to mitigate the impact of pelvic fracture on individual and population health.

## Data Availability

The original contributions presented in the study are included in the article/Supplementary material, further inquiries can be directed to the corresponding author.

## Supplementary material

Supplementary Table 1 The number of incidence cases and the age-standardized incidence rate of fracture of pelvis in 1990 and 2021, and its trends from 1990 to 2021 globally.

Supplementary Table 2 The number of prevalence cases and the age-standardized prevalence rate of fracture of pelvis in 1990 and 2021, and its trends from 1990 to 2021 globally.

Supplementary Table 3 The number of YLDs cases and the age-standardized YLDs rate of fracture of pelvis in 1990 and 2021, and its trends from 1990 to 2021 globally.

Supplementary Table 4 The predicted results in the fracture of pelvis-related numbers and age-standardized rates of incidence, prevalence, and YLDs by sex globally from 2022 to 2046 of the APC model.

Supplementary Table 5 The predicted results in the fracture of pelvis-related numbers and age-standardized rates of incidence, prevalence, and YLDs by sex globally from 2022 to 2046 of the BAPC model.
