# Supplementary material for: Global, regional, and national burden of fracture of pelvis, 1990–2021: analysis of data from the Global Burden of Disease Study 2021
Source: Front Public Health. 2025 Jun 18;13:1610604. doi: 10.3389/fpubh.2025.1610604 (PMC12213497; doi:10.3389/fpubh.2025.1610604)

**Global, regional, and national burden of fracture of pelvis, 1990-2021: analysis of data from the global burden of disease study 2021**

**Figure legends**

**Figure S1.** Numbers and age-standardized rates of fracture of pelvis-related incidence, prevalence, and YLDs for both sexes in 2021. Abbreviations: YLDs, disability-adjusted life years.

**Figure S2.** Numbers and age-standardized rates of fracture of pelvis-related incidence, prevalence, and YLDs for different age groups in 2021. Abbreviations: YLDs, disability-adjusted life years.

**Figure S3.** Numbers and age-standardized rates of fracture of pelvis-related incidence, prevalence, and YLDs for different SDI regions in 2021. Abbreviations: YLDs, disability-adjusted life years.

**Figure S4.** Numbers and age-standardized rates of fracture of pelvis-related incidence, prevalence, and YLDs for different GBD regions in 2021. Abbreviations: YLDs, disability-adjusted life years.

**Figure S5.** Trends in the numbers and age-standardized rates of fracture of pelvis incidence, prevalence, and YLDs globally from 1990 to 2021. Abbreviations: YLDs, years lived with disability.

**Figure S6.** Trends in the numbers and age-standardized rates of fracture of pelvis-related incidence, prevalence, and YLDs globally by sexes from 1990 to 2021. Abbreviations: YLDs, years lived with disability.

**Figure S7.** Trends in the numbers and age-standardized rates of fracture of pelvis-related incidence, prevalence, and YLDs globally by age groups from 1990 to 2021. Abbreviations: YLDs, years lived with disability.

**Figure S8.** Trends in the numbers and age-standardized rates of fracture of pelvis-related incidence, prevalence, and YLDs globally by SDI regions from 1990 to 2021. Abbreviations: YLDs, years lived with disability.

**Figure S9.** The EAPC value for the age-standardized incidence, prevalence, and YLDs rates of fracture of pelvis from 1990 to 2021. Abbreviations: EAPC, estimated annual percentage change; YLDs, years lived with disability.

**Figure S1.** Numbers and age-standardized rates of fracture of pelvis-related incidence, prevalence, and YLDs for both sexes in 2021. Abbreviations: YLDs, disability-adjusted life years.


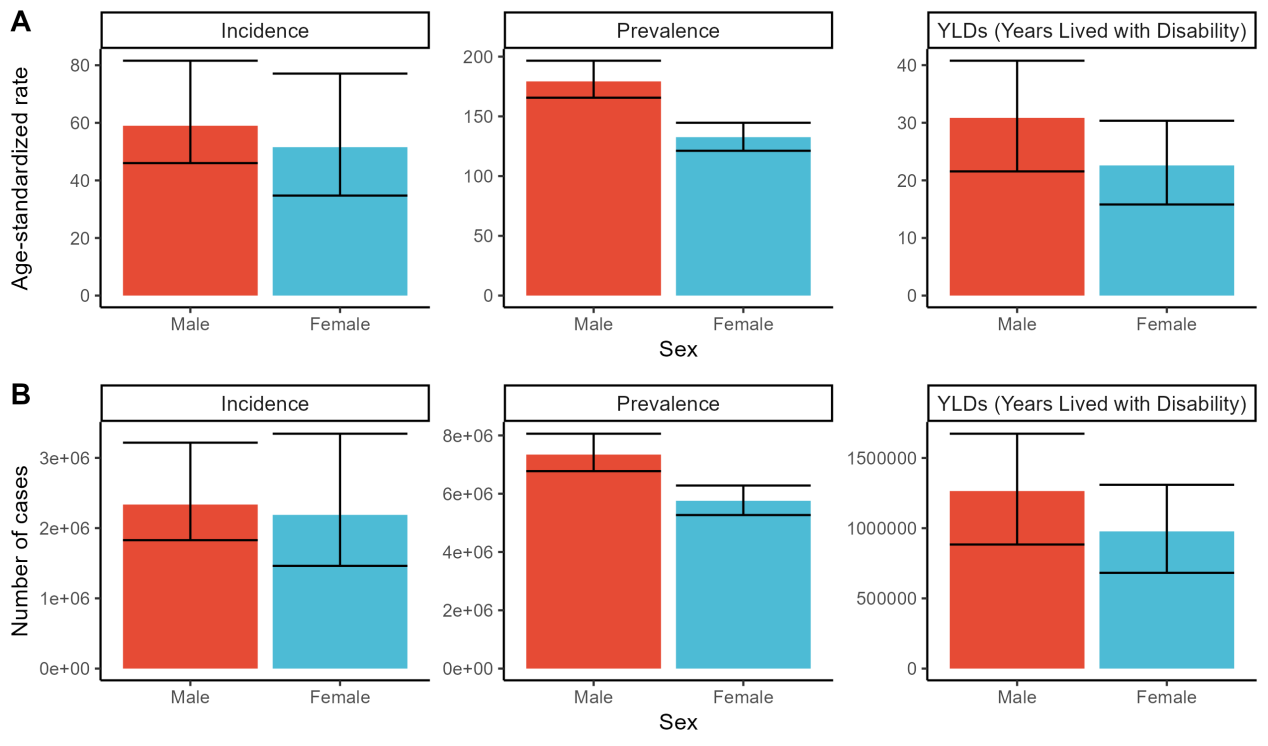


**Figure S2.** Numbers and age-standardized rates of fracture of pelvis-related incidence, prevalence, and YLDs for different age groups in 2021. Abbreviations: YLDs, disability-adjusted life years.


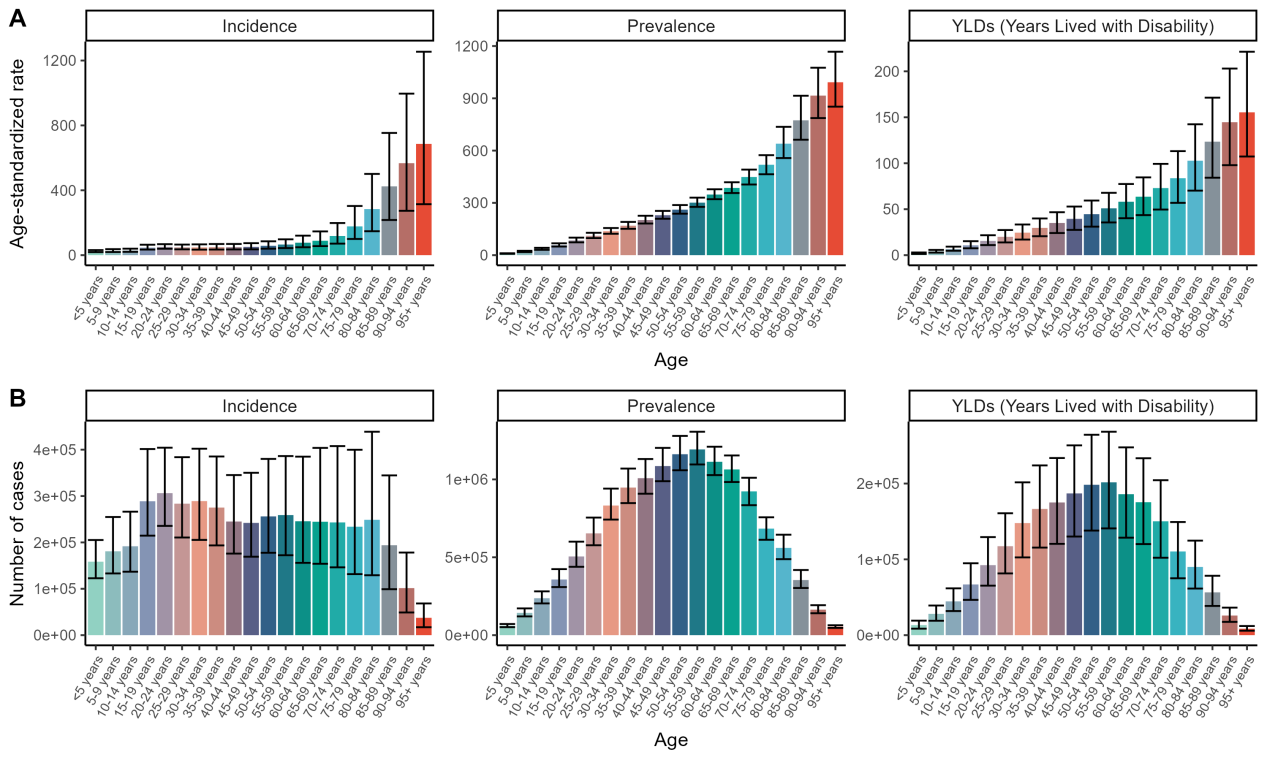


**Figure S3.** Numbers and age-standardized rates of fracture of pelvis-related incidence, prevalence, and YLDs for different SDI regions in 2021. Abbreviations: YLDs, disability-adjusted life years.


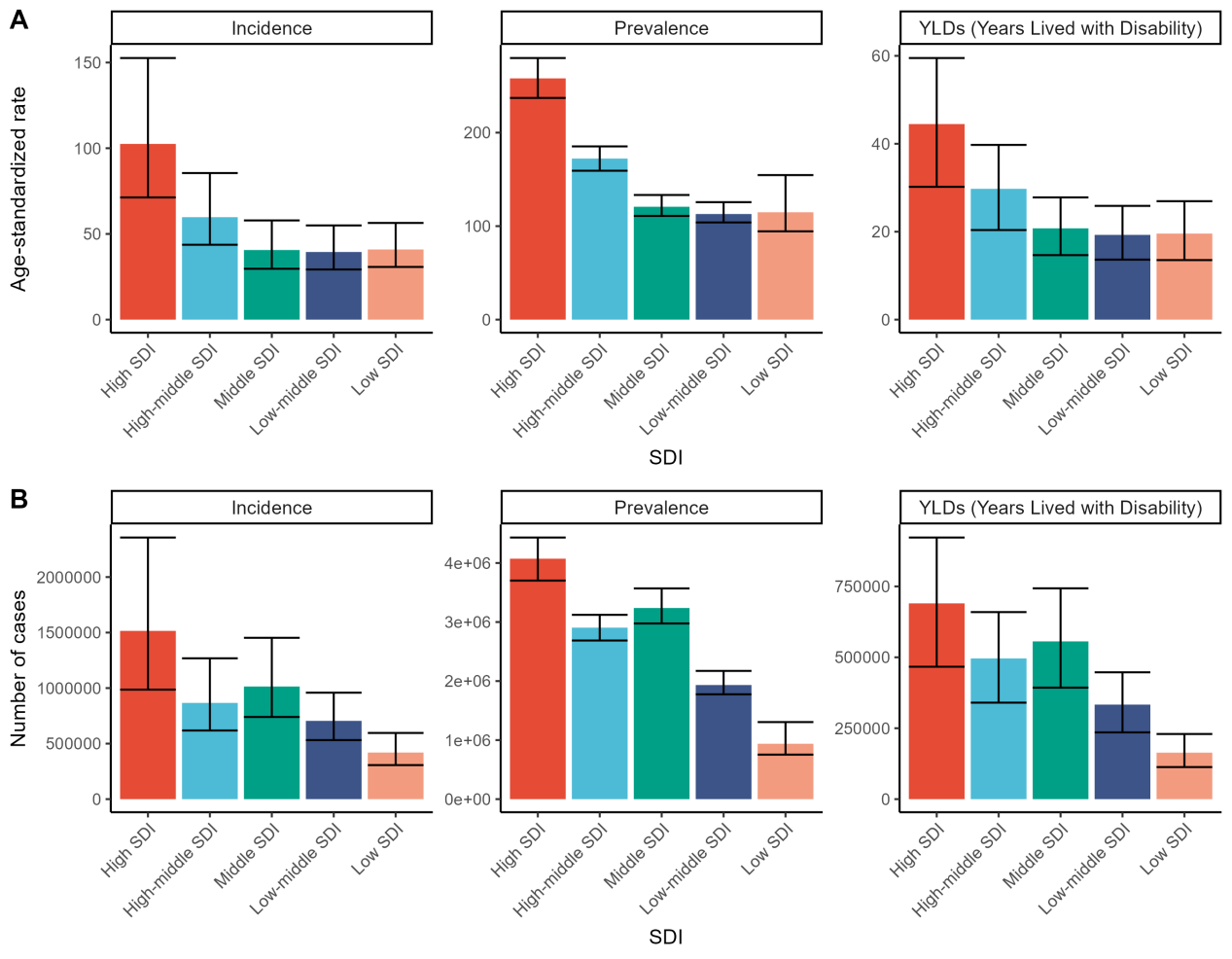


**Figure S4.** Numbers and age-standardized rates of fracture of pelvis-related incidence, prevalence, and YLDs for different GBD regions in 2021. Abbreviations: YLDs, disability-adjusted life years.


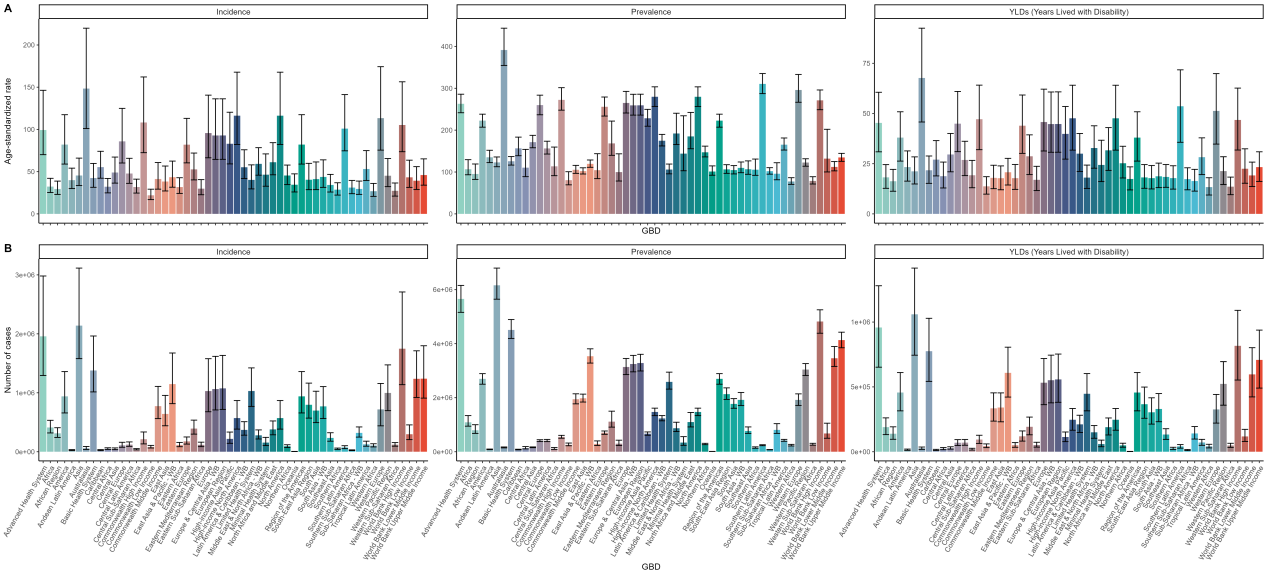


**Figure S5.** Trends in the numbers and age-standardized rates of fracture of pelvis incidence, prevalence, and YLDs globally from 1990 to 2021. Abbreviations: YLDs, years lived with disability.


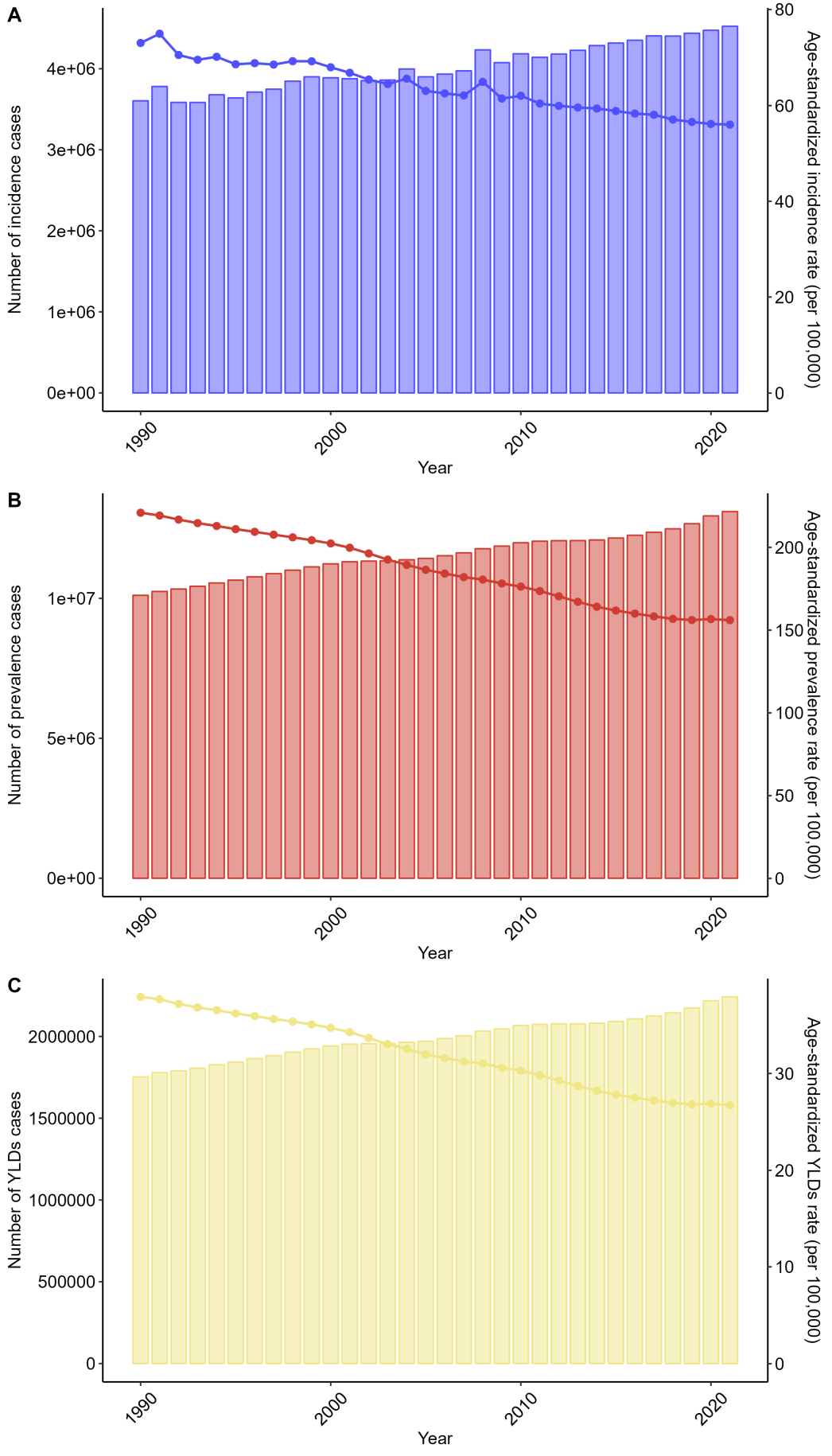


**Figure S6.** Trends in the numbers and age-standardized rates of fracture of pelvis-related incidence, prevalence, and YLDs globally by sexes from 1990 to 2021. Abbreviations: YLDs, years lived with disability.


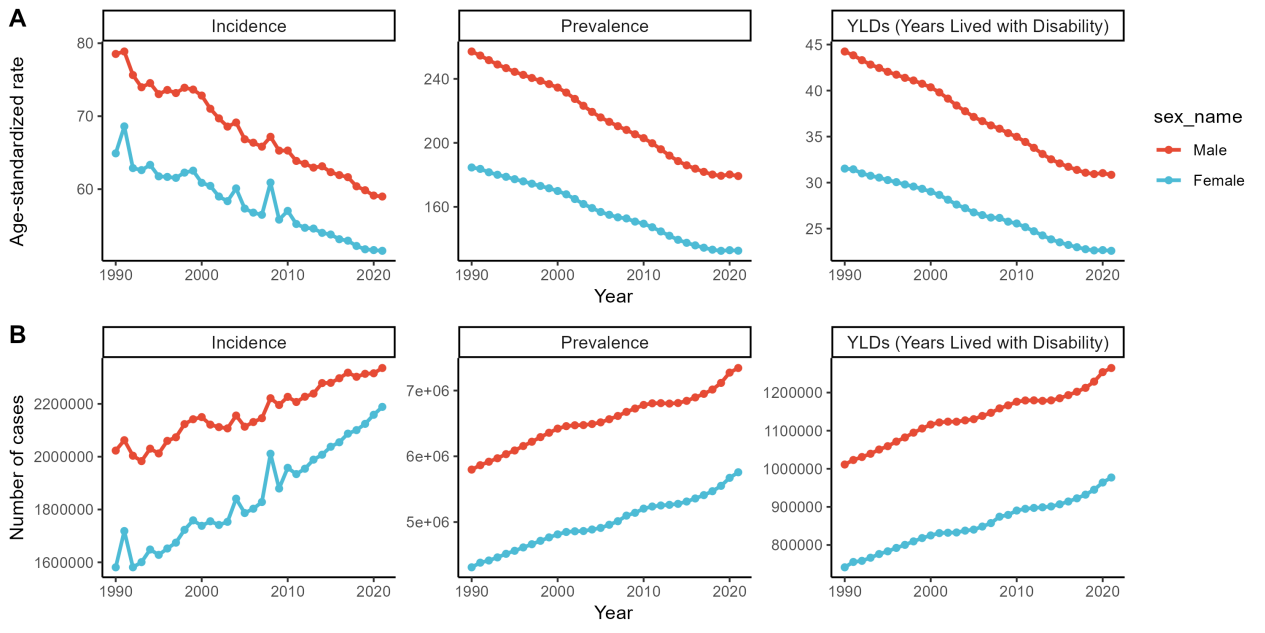


**Figure S7.** Trends in the numbers and age-standardized rates of fracture of pelvis-related incidence, prevalence, and YLDs globally by age groups from 1990 to 2021. Abbreviations: YLDs, years lived with disability.


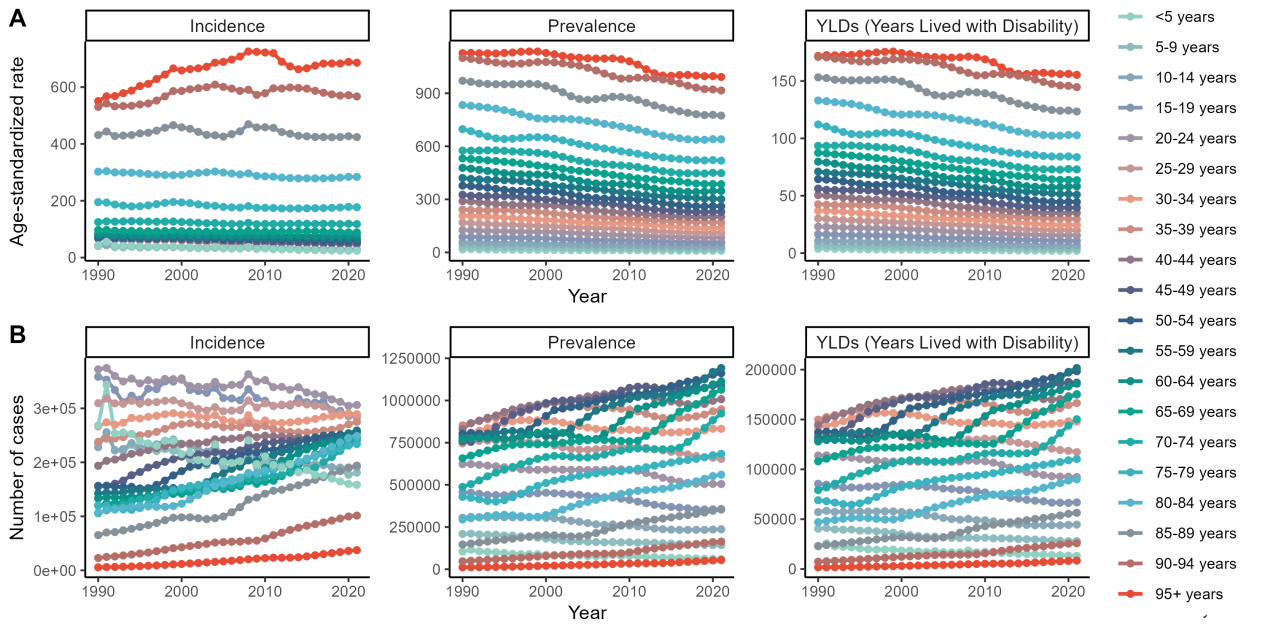


**Figure S8.** Trends in the numbers and age-standardized rates of fracture of pelvis-related incidence, prevalence, and YLDs globally by SDI regions from 1990 to 2021. Abbreviations: YLDs, years lived with disability.


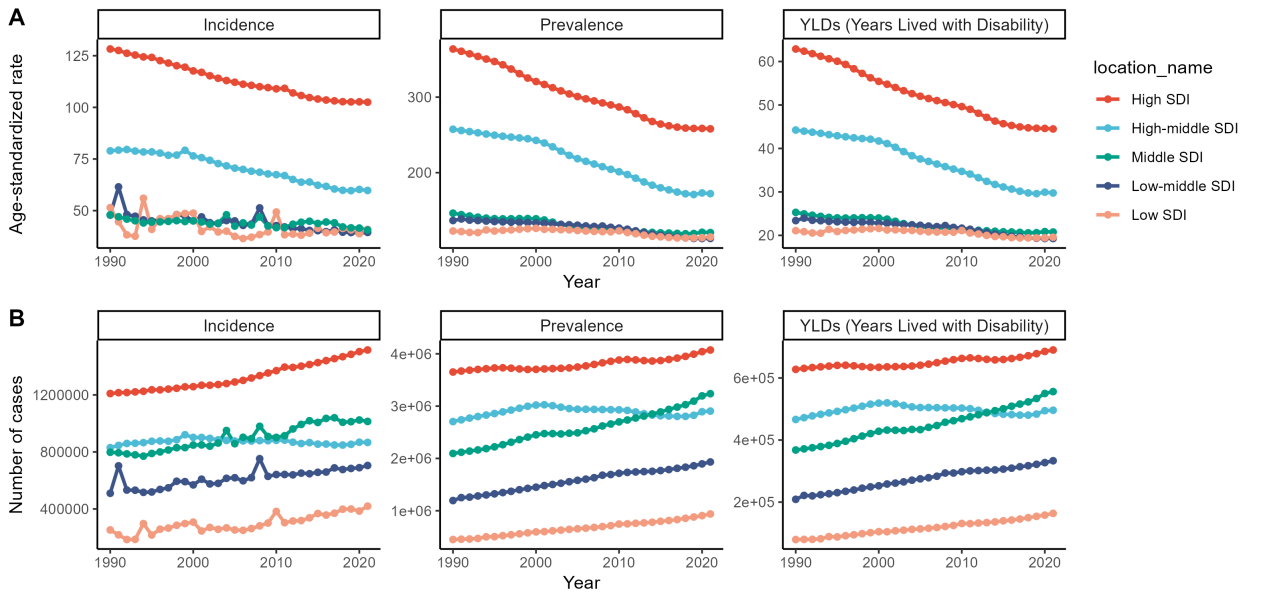


**Figure S9.** The EAPC value for the age-standardized incidence, prevalence, and YLDs rates of fracture of pelvis from 1990 to 2021. Abbreviations: EAPC, estimated annual percentage change; YLDs, years lived with disability.


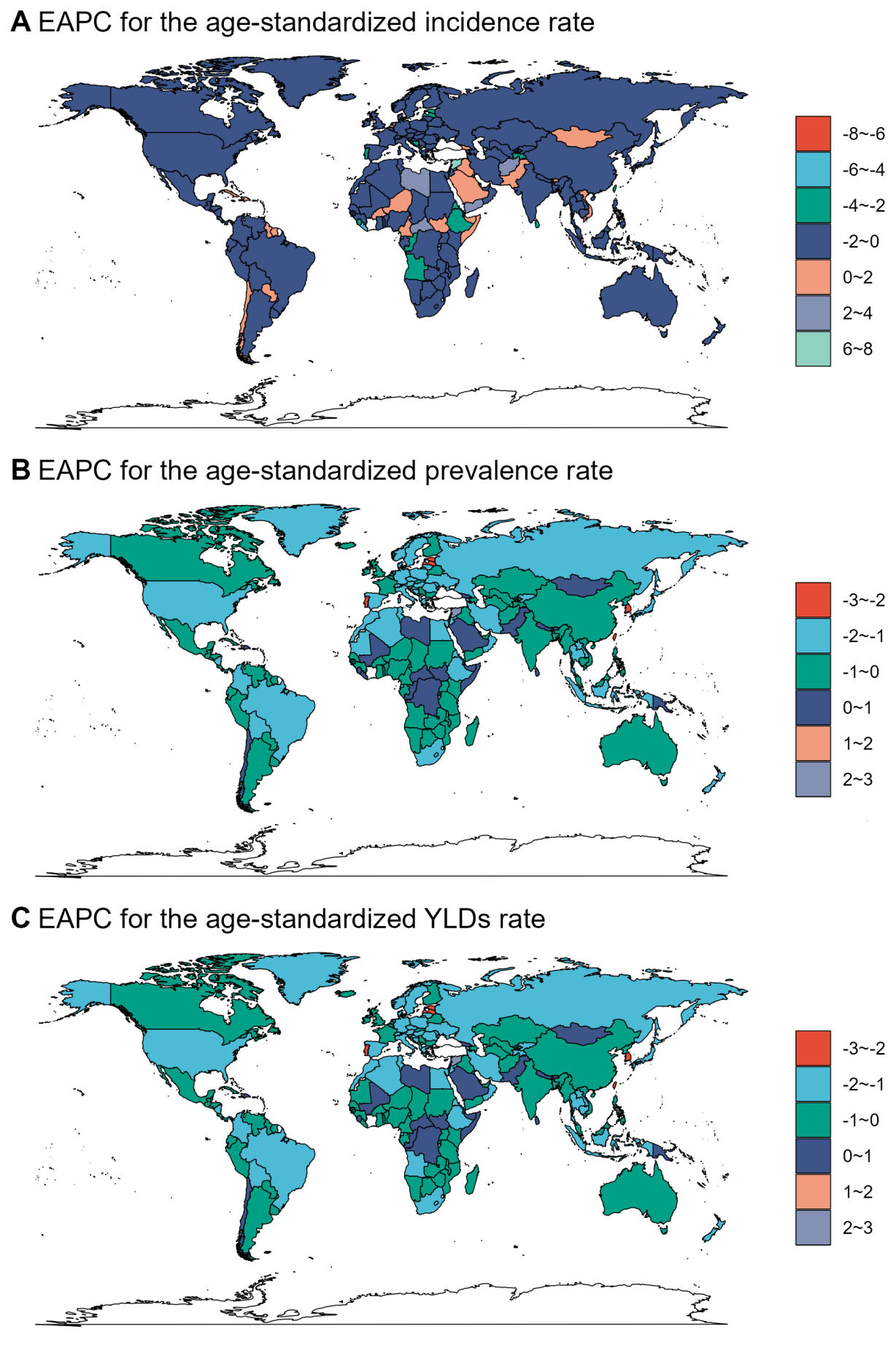

Supplement: Supplementary file 6 [file Data_Sheet_1.docx]
